# A Case of Surgical Revascularization in a Patient with Uncontrolled Renovascular Hypertension and Renal Dysfunction after Repeated Percutaneous Transluminal Renal Angioplasty (PTRA) for More Than 10 Years

**DOI:** 10.3400/avd.cr.25-00022

**Published:** 2026-03-19

**Authors:** Yasutake Momokawa, Koji Maeda

**Affiliations:** Department of Vascular Surgery, International University of Health and Welfare, Narita Hospital, Narita, Chiba, Japan

**Keywords:** PTRA, ISR, aorto-renal artery bypass

## Abstract

Percutaneous transluminal renal angioplasty (PTRA) is a treatment for renovascular hypertension due to renal artery stenosis. However, postoperative complications in stent re-stenosis/occlusion may occur frequently. A 60-year-old male patient presented to our hospital with uncontrolled hypertension and a deterioration of renal function. He had undergone an initial renal stenting 10 years earlier, followed by repeat PTRAs during follow-up for in-stent restenosis. The left renal stent was found to be completely occluded, while the right renal stent was found to be 75% stenosed. We performed an aorto-renal artery bypass. The bypass was patent without stenosis and the renovascular hypertension was recovered.

## Introduction

Renal artery stenosis (RAS) is considered as one of the most common causes of secondary hypertension and renal dysfunction. It is also frequently associated with heart failure due to activation of the renin-angiotensin-aldosterone system.

Treatment includes medical therapy, percutaneous transluminal renal angioplasty (PTRA), and surgical revascularization. PTRA has been reported to be effective in renal vascular hypertension that is resistant to medical therapy.^1,2)^ However, in-stent restenosis (ISR) is one of the risks of stenting, which is estimated to be approximately 10%–20%.^3–5)^ Risk factors for ISR include small vessel diameter, treatment for stent restenosis, stent type, smoking, and elevated baseline peak systolic velocity on Doppler ultrasound.^6,7)^ On the other hand, surgical revascularization has been shown to have good long-term outcomes with a low incidence of complications.^8)^ In this study, we report the successful results of an aorto-renal artery bypass in a patient who developed renovascular hypertension, renal function deterioration, and heart failure due to ISR after repeated PTRAs.

## Case Report

Informed consent was obtained from the patient for publication of the case, details, and images.

The case is a 60-year-old male who was referred to our hospital for the treatment of renal artery ISR. In his early 50s, he developed angina pectoris and was admitted to treatment. During percutaneous coronary intervention (PCI), he was diagnosed with bilateral RAS and underwent bilateral PTRA. During a routine coronary angiography examination 6 months later, ISR of the renal artery was also observed, and additional PCI and PTRA were performed.

**Fig. 1:**
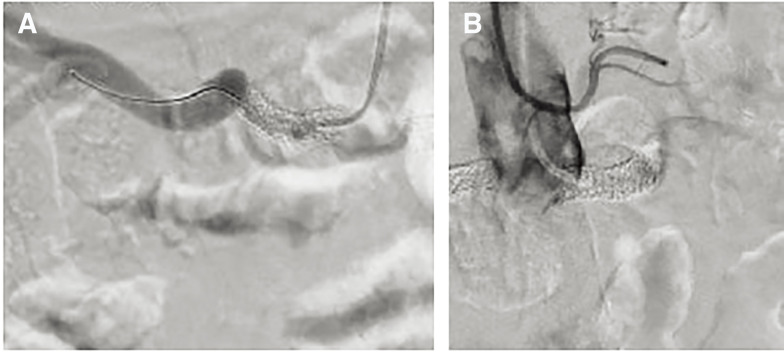
Angiography showed severe in-stent restenosis of the right renal stent (**A**) and occlusion of the left renal artery stent (**B**).

Finally, he underwent 6 PTRAs over a 10-year period, including 3 stents for the right renal artery (2 of Express stents; Boston Scientific, Natick, MA, USA, and 1 of Nobori stent; Terumo, Tokyo, Japan) and 2 stents for the left renal artery (Express stent and Xience stent; Abbott Vascular, Santa Clara, CA, USA). In his 60s, he was transferred to another hospital, and he underwent coronary angiography for recurrent heart failure. The coronary artery stents were patent, but angiography showed occlusion of the left renal artery stent and recurrent ISR of the right renal artery stent (**Fig. 1**). Cardiologists attempted PTRA but were unable to pass the wire into the stenotic lesions. Therefore, the patient was referred to our hospital due to deterioration of renal function and heart failure associated with renovascular hypertension.

The patient was taking 3 antihypertensive medications, including a calcium antagonist, a β-blocker, and an angiotensin II receptor blocker, and his blood pressure was 162/78 mmHg. Abdominal bruit was not identified. Comorbidities include dyslipidemia, diabetes mellitus on insulin therapy, and multiple PCIs for coronary artery disease. He previously smoked 60 cigarettes per day but quit 15 years ago.

The preoperative Doppler ultrasound showed that the right kidney was 116 mm in length with an resistive index (RI) of 0.76, and the left kidney was 102 mm with an RI of 0.46. However, after multiple PTRA procedures, the left renal artery was found to be occluded several months later.

Blood examinations showed a high level of brain natriuretic peptide (BNP) 1040 pg/mL, and low renal function (creatinine (Cre) 1.57 mg/dL and eGFR 36.6 mL/min).

We decided to reconstruct only the right renal artery because an atrophic change was identified in the left kidney.

**Fig. 2:**
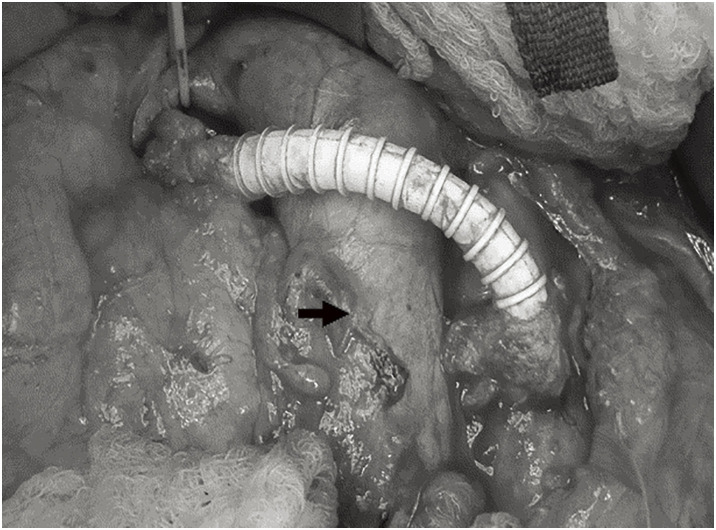
The bypass route was anterior to the inferior vena cava (arrow). The graft had an end-to-side anastomosis with the aorta and an end-to-end anastomosis with the renal artery.

The operation was performed under general anesthesia. A 4-Fr sheath was inserted into the common femoral artery for intraoperative angiography. A catheter was placed in the orifice of the renal artery via the 4-Fr sheath. Angiography showed occlusion of the left renal artery stent and approximately 75% stenosis of the right renal artery stent Laparotomy was performed with a midline abdominal incision, and the great saphenous vein was harvested from the left leg. The right renal artery was identified using the Kocher’s maneuver, and the right renal vein was exposed from the right side of the vena cava. The abdominal aorta was then exposed to allow clamping. Since the dilated great saphenous vein was unsuitable due to inadequate caliber, a 6-mm Propaten graft (W.L.Gore & Associates, Flagstaff, AZ, USA) was used for the bypass graft. After systemic heparinization (60 U/kg), the proximal aortic clamp was applied just below the origin of the inferior mesenteric artery, and the distal clamp was placed above the aortic bifurcation. A 10-mm aortotomy was created on the anterior wall of the aorta approximately 2 cm distal to the origin of the inferior mesenteric artery. The graft was then anastomosed to the aorta in an end-to-side fashion using continuous 5–0 polypropylene sutures. Subsequently, the right renal artery was transected, and an end-to-end anastomosis between the renal artery and the graft was performed using 6–0 polypropylene without renal protective perfusion (**Fig. 2**).

**Fig. 3:**
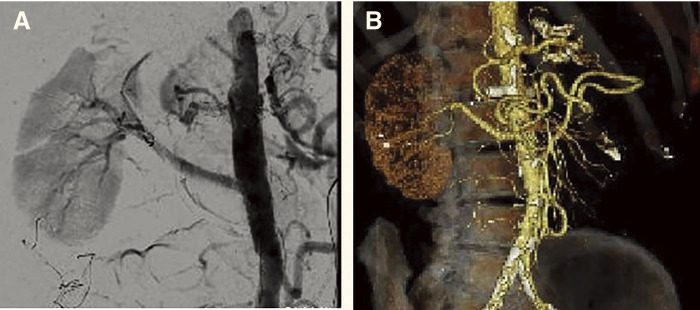
The aorto-renal bypass was patent on intraoperative angiography (**A**), and the right renal artery was well-contrasted in the peripheral region on postoperative CTA (**B**). CTA: computed tomography angiography

Completion angiography showed excellent blood flow in the bypass without peripheral embolization to the kidney (**Fig. 3**).

The operative time was 205 min, the blood loss was 180 mL, and the time of right renal artery occlusion was 17 min, respectively.

The postoperative course was uneventful, and the blood pressure decreased to 112/86 mmHg postoperatively.

Postoperative CT at 3 years after surgery showed a patent bypass with no deterioration in renal function (Cre 1.57 mg/dL, eGFR 36.6 mL/min), and no cardiovascular events, including heart failure, were observed after the surgery. However, postoperative Doppler ultrasound showed that the right kidney was 112 mm in length with RI of 0.76. In addition, the patient was able to reduce antihypertensive medication to one medication.

## Discussion

RAS is often caused by atherosclerosis. PTRA has become more common because it is easy to perform, but the results of randomized controlled trials (RCTs) have shown no difference in improvement, including renal function, between medical therapy and PTRA.^1,2)^ In addition, there is some risk of restenosis. According to the recent guidelines,^3)^ PTRA is recommended as a definite or possible indication for high-risk atherosclerotic renovascular disease, such as those with progressive chronic kidney disease in high-grade [>75%] RAS with bilateral or solitary kidney, and intolerance to angiotensin-converting enzyme inhibitors or angiotensin II receptor blockers. By contrast, surgical revascularization is generally reserved for rare cases, such as fibromuscular dysplasia unresponsive to angioplasty, or complex vascular anatomy unsuitable for PTRA. Stone et al.^4)^ reported a target vessel revascularization rate of 10.6% at 10 years, and all patients were treated again with PTRA, with 20% of patients requiring repeat treatment. When restenosis occurs, uncontrollable hypertension and renal dysfunction have been observed in over 60% of patients.^4)^ In addition, stents have been reported to have better patency rates than PTRA in the treatment of ISR, and drug-eluting stents are also used in renal artery stenosis. However, ISR occurred in 25% of patients.^6)^

Risk factors for ISR include various parameters such as age, small vessel diameter, stent size, stent type, smoking smaller kidney, and high RI on Doppler ultrasound.^6,7)^

Steuer et al.^8)^ reported 40 cases of renal artery revascularization for the treatment of renal artery stenosis and renal aneurysm. Of these, 31 patients had renal artery stenosis, 25 had atherosclerotic disease, and 8 had fibromuscular dysplasia (FMD). Of the 25 patients, 15 patients underwent aorto-renal artery bypass, 2 patients underwent ilio-renal artery bypass, and 8 patients underwent endarterectomy. One patient died during surgery, and one patient required hemodialysis. All patients could have a decrease in blood pressure and reduce antihypertensive medication. The 30-day mortality after surgical revascularization is approximately 4%, and the factors of advanced age or heart failure are associated with a poor prognosis.^8)^ A systematic review comparing surgical revascularization and PTRA article showed that surgical revascularization was better than EVT in terms of improving blood pressure and renal function.^9)^ Regarding graft selection, there is a lack of RCTs comparing vein grafts and prosthetic grafts. However, some reports indicate that approximately half of the grafts used in aorto-renal bypass were prosthetic grafts.^10)^

In our case, repeated PTRAs were performed for recurrent ISR, and coronary stents were used to treat ISR. Because the size of coronary stents is generally smaller than that of renal stents, additional PTRA was considered inappropriate for the next step. In addition, the left stent was completely occluded, and the right renal artery stent also showed 75% stenosis with renal dysfunction. For these reasons, we decided to perform surgical revascularization. Although it is difficult to decide when to switch to open surgery after PTRA, we believe that it should be considered in case of a solitary kidney, ISR after repeated PTRA, and the presence of small-diameter stent such as a coronary stent.

## Conclusions

Surgical revascularization has been successfully performed for renal artery ISR complicated by renovascular hypertension and heart failure. We believe that surgical revascularization is one of the effective options for repeated ISR after PTRA.
